# Non-mitogenic FGF2 protects cardiomyocytes from acute doxorubicin-induced toxicity independently of the protein kinase CK2/heme oxygenase-1 pathway

**DOI:** 10.1007/s00441-018-2905-z

**Published:** 2018-08-29

**Authors:** Navid Koleini, Barbara E. Nickel, Andrea L. Edel, Robert R. Fandrich, Amir Ravandi, Elissavet Kardami

**Affiliations:** 1 Institute of Cardiovascular Sciences, St. Boniface Hospital Albrechtsen Research Centre., 351 Tache Ave, Winnipeg, Manitoba R2H2A6 Canada; 2 Department of Physiology and Pathophysiology, University of Manitoba, Winnipeg, Canada; 3 Department of Human Anatomy and Cell Sciences, University of Manitoba, Winnipeg, Canada; 4 Interventional Cardiology, Section of Cardiology, Max Rady College of Medicine, University of Manitoba, Winnipeg, Canada

**Keywords:** Non-mitogenic FGF2, Acute doxorubicin cardiotoxicity, CK2-HO-1 signaling, Oxidized phospholipids, Rat cardiomyocytes

## Abstract

Doxorubicin (Dox)-induced cardiotoxicity, a limiting factor in the use of Dox to treat cancer, can be mitigated by the mitogenic factor FGF2 in vitro, via a heme oxygenase 1 (HO-1)-dependent pathway. HO-1 upregulation was reported to require protein kinase CK2 activity. We show that a mutant non-mitogenic FGF2 (S117A-FGF2), which does not activate CK2, is cardioprotective against acute cardiac ischemic injury. We now investigate the potential of S117A-FGF2 to protect cardiomyocytes against acute Dox injury and decrease Dox-induced upregulation of oxidized phospholipids. The roles of CK2 and HO-1 in cardiomyocyte protection are also addressed.

Rat neonatal cardiomyocyte cultures were used as an established in vitro model of acute Dox toxicity. Pretreatment with S117A-FGF2 protected against Dox-induced: oxidative stress; upregulation of fragmented and non-fragmented oxidized phosphatidylcholine species, measured by LC/MS/MS; and cardiomyocyte injury and cell death measured by LDH release and a live-dead assay. CK2 inhibitors (TBB and Ellagic acid), did not affect protection by S117A-FGF2 but prevented protection by mitogenic FGF2. Furthermore, protection by S117A-FGF2, unlike that of FGF2, was not prevented by HO-1 inhibitors and S117A-FGF2 did not upregulate HO-1. Protection by S117A-FGF2 required the activity of FGF receptor 1 and ERK.

We conclude that mitogenic and non-mitogenic FGF2 protect from acute Dox toxicity by common (FGFR1) and distinct, CK2/HO-1- dependent or CK2/HO-1-independent (respectively), pathways. Non-mitogenic FGF2 merits further consideration as a preventative treatment against Dox cardiotoxicity.

**Electronic supplementary material:**

The online version of this article (10.1007/s00441-018-2905-z) contains supplementary material, which is available to authorized users.

## Introduction

Doxorubicin (Dox) is a chemotherapy drug that is widely used to treat many types of malignancies including breast cancer, lymphoma and acute lymphoblastic leukemia in patients of all ages; cardiotoxicity that can manifest acutely, sub-acutely, or late-onset has posed limitation to the use of Dox (Zamorano et al. 2016). Dox cardiotoxicity is attributed to elevated oxidative stress, DNA and mitochondrial damage and deleterious changes in gene expression, culminating in the dysregulation of multiple signal transduction pathways leading to cardiac cell death (Koleini and Kardami 2017). Currently, the only FDA-approved drug to mitigate Dox-induced cardiotoxicity is dexrazoxane, which has been of restricted use due to the concern for cancer outcomes (van Dalen et al. 2011).There is clearly a need for new approaches to protect the heart, without compromising the efficacy of, Dox. Fibroblast growth factor 2 (FGF2) is a heparin-binding growth factor expressed in all tissues as high (> 20 kDa) and low (18 kDa) molecular weight isoforms (Kardami et al. 2007; Santiago et al. 2011; Santiago et al. 2014). Please note that for the rest of this work the term FGF2 will refer to the 18-kDa FGF2 isoform.

We have recently published that FGF2 protects neonatal rat cardiomyocytes against acute Dox-induced damage and cell death by a mammalian target of rapamycin/nuclear factor (erythroid-derived 2)-like (Nrf2)/heme oxygenase−1 (HO-1)-dependent pathway in vitro (Koleini et al. 2017). A concern with using FGF2 as a cardioprotective strategy against Dox is the possibility of undesirable effects on cancer cell proliferation and survival. FGF2 is a potent mitogen and angiogenic agent and may increase tumor proliferation and blood vessel formation (Jiang et al. 2004; Li et al. 2016). Previous studies have demonstrated that a mutated form of FGF2, carrying a serine-to-alanine (S117A) substitution (S117A-FGF2) retains acute cardioprotective potential against cardiac ischemic injury but lacks mitogenic and angiogenic activity (Bailly et al. 2000; Jiang et al. 2002; Jiang et al. 2004). It is postulated that if the S117A-FGF2 retains cardioprotective properties against Dox-induced cardiomyocyte oxidative stress and injury/death, it would merit further consideration as a prophylactic treatment against Dox.

The S117A-FGF2 has lost the ability to interact with and activate the ubiquitous protein kinase CK2 that promotes cell proliferation and angiogenesis (Bailly et al. 2000; Feng et al. 2012). Furthermore, CK2 is required for the upregulation of HO-1 in chondrocytes (Kim et al. 2012), which would suggest that S117A-FGF2 may be incapable of upregulating HO-1 in cardiomyocytes. Since HO-1 was required for the protective effect of FGF2 (Koleini et al. 2017), the possibility arose that S117A-FGF2 may not protect against Dox-induced cardiomyocyte damage, or, if it did, a non-CK2/HO-1 pathway would be involved.

Here, we will report on our findings regarding the ability of S117A-FGF2 to protect cardiomyocytes against Dox damage, increased oxidative stress, increased oxidized phosphatidylcholines and the role of CK2/HO-1 and ERK in cardioprotection by S117A-FGF2.

## Materials and methods

This study was done according to the NIH Guide for the Care and Use of Laboratory Animals (NIH Publication, 8th Edition. Revised 2011). Approval was received from the Protocol Management and Review Committee of the University of Manitoba.

### Cultures

Hearts were collected from 1- to 2-day-old rat pups and ventricular cardiomyocytes were isolated as previously described (Koleini et al. 2017). After isolation, cardiomyocytes were plated at a density of 5 × 10^4^ cells/cm^2^ on collagen-coated culture plates in 20% fetal bovine serum (FBS) F10 media and allowed to attach overnight. The next day culture medium was changed to 0.5% FBS, 1% insulin, 1% transferrin/selenium, 0.5% ascorbic acid and 1% bovine serum albumin and cells were incubated for 48 h. S117A-FGF2 (10 ng/ml) was added to the cultures 30 min before Dox addition. Myocytes were then exposed to Dox (0.5 μM) for up to 24 h. Inhibitors were added 30 min before the addition of S117A-FGF2. Culture media was collected and stored at − 80°C for LDH assays. The cells were snap frozen in liquid nitrogen and stored at − 80°C for extraction and western blotting.

### Reagents

Recombinant rat FGF2 and S117A-FGF2 were produced in-house using plasmids described in (Bailly et al. 2000; Koleini et al. 2017). Histidine Tag at the N-terminal of the fusion proteins was used to purify the recombinant proteins using Nickel-sepharose (Ni-sepharose, high performance from GE healthcare, # 17-5268-01), according to the manufacturers protocols. FGFR1 antibody (anti-pY766-FGFR1) was purchased from SantaCruz (#sc-16309). The phospho (P) ERK (#9101), ERK (#9102), AKT (#9272), P-AKT (#9271), P38 (#9212) and P-P38 (#9211S) antibodies were purchased from Cell Signaling Technology. Optima LC/MS grade solvents were obtained from Fisher Scientific (Ottawa, Ontario, Canada). 1,2-Dinonanoyl-sn-glycero-3-phosphocholine (09:0 PC) was purchased from Avanti Polar Lipids (Alabaster, AL, USA). Butylated hydroxytoluene (BHT) was purchased from Sigma-Aldrich (Oakville, Ontario, Canada).

### Calcein-AM/ethidium homodimer viability assay

Cells were rinsed twice with phosphate buffered saline (PBS) at 37 °C, then incubated with Calcein-AM (2 μM, C3100, Thermofisher) and ethidium homodimer (2.5 μM, E1169, Thermofisher) in PBS for 30 min. Images were taken using an LSM 5 PASCAL fluorescence microscope.

### MTT assay

MTT (3-(4,5-dimethylthiazol-2-yl)-2,5-diphenyltetrazolium bromide (Sigma-Aldrich, # M2128) assay was used to measure cell number. MCF-7 cells were grown in 24 well plates (1 ml of 0.5% FBS, 1% insulin, 1% transferrin/selenium, 0.5% ascorbic acid and 1% bovine serum in DEM) until 50% confluence. FGF2 or S117A-FGF2 (10 ng/ml) were added to the cultures for 24 h. The next day 100 μl of 5 mg/ml MTT in PBS was added to the cultures for 1 h. The media was discarded and cells were washed gently in phosphate buffered saline (PBS) twice. One hundred fifty microliters of 1:1 ethanol-DMSO were added to each well and mixed for 10 min. The absorbance of formazen was read at 570 nm; the absorbance at 630 nm was used as the reference.

### Protein (western) immunoblotting

Cells were scraped in sodium dodecyl sulphate (SDS)/polyacrylamide gel electrophoresis (PAGE) sample buffer (1%(*w*/*v*) SDS) supplemented (1:100) with protease inhibitor cocktail (Sigma-Aldrich, #8304) and phosphatase inhibitor cocktail set II and IV (Calbiochem, #524625 and #524628). The lysates were boiled, sonicated and centrifuged (14,000×*g* for 15 min) to remove debris. Bicinchoninic acid (BCA) assay was used to determine protein concentration in the samples. Following SDS-PAGE, proteins were transferred to polyvinylidene fluoride (PVDF) membranes. 0.01% (*w*/*v*) Ponceau S (Sigma-Aldrich, P3504) in 0.15% trichloroacetic acid was used to stain the membrane to assess protein transfer and used to correct for protein loading. The membranes were incubated in 5% BSA/TBS-T for 1 h at room temperature to block non-specific binding.

### Commercial kit-based assays

The Pierce™ LDH Cytotoxicity Assay Kit (Thermofisher, 88953) was used to measure LDH in supernatants, as an indicator of plasma membrane damage. To measure the levels of ROS, DCF-DA (2′7′-dichlorodihydrofluorescein diacetate, Thermofisher, D399) was used. The assay was done according to the manufacturer's protocols.

### Phospholipid extraction from cardiomyocytes

Cardiomyocytes were grown on 60-mm dishes. After the cells were treated, they were scraped into 200-μl PBS-EDTA. The lysates were quickly frozen and kept at − 80 °C. Thawed cardiomyocytes were lysed using sonication. One hundred and fifty microliters of cell mixture were extracted with 2:1 (vol/vol) chloroform:methanol containing 0.01% BHT using the method described by Folch et al. (Folch et al. 1957). One hundred microliters of 1,2-dinonanoyl-*sn*-glycero-3-phosphocholine (09:0 PC; 0.1 μg/ml) were added as internal standard prior to lipid extraction. Twenty microliters of the lysates were used to determine protein concentration. To solubilize the proteins, 5 μl of the 5× SDS buffer was added to the lysates. The mixtures were briefly boiled and sonicated. Protein concentration was measured using the BCA method.

### Oxidized phosphatidylcholine (OxPC) analysis by LC/MS/MS

Reverse phase-high-performance liquid chromatography (RP-HPLC) was used to separate oxidized phosphatidylcholine (OxPC) species, as previously described (Ganguly et al. 2018). Cardiomyocyte lipid extracts were reconstituted in 100 μL of mobile phase A. Thirty microliters were injected onto an Ascentis Express C18 HPLC column (15 cm × 2.1 mm, 2.7 μm; Supelco Analytical, Bellefonte, Pennsylvania, USA) using a Prominence HPLC system (Shimadzu Corporation, Canby, Oregon, USA). Analyte separation was accomplished using a linear gradient of solvents A (acetonitrile/water, 60:40 vol/vol) and B (isopropanol/acetonitrile, 90:10, vol/vol), each containing 10 mM ammonium formate and 0.1% formic acid. The elution profile was as follows: initial solvent B at 32%, increased to 45% B until 4.00 min; 5.00 min 52% B; 8.00 min 58% B; 11.00 min 66% B; 14.00 min 70% B; 18.00 min 75% B; 21.00 min 97% B; 25.00 min 97% B; 25.10 min 32% B with an overall run time of 30.10 min. Separation was performed using a flow rate of 0.26 ml/min, while the temperature of the sample tray and column oven were maintained at 4 and 45 °C, respectively.

Analyte detection was accomplished using a 4000 QTRAP® triple quadrupole mass spectrometer (MS) (AB Sciex, Framingham, MA, USA) as has been fully described (Ganguly et al. 2018; Torzewski et al. 2017). OxPC were calculated relative to the amount of internal standard with final results represented as amount (ng) of OxPC per microgram protein.

### Data analysis and statistics

Each experiment was repeated at least three times using different cardiomyocyte cultures. In each repeat, the group size was 3–5. GraphPad Prism 6 was used for statistical analysis. One-way (Tukey post hoc or LSD) or two-way ANOVA (sidak post hoc or LSD) was used (as appropriate). *P* value was set at *P* < 0.05. The data are shown as mean ± standard error mean (SEM).

## Results

### The effect of S117A-FGF2 against Dox-induced cardiomyocyte damage

**Fig. 1:**
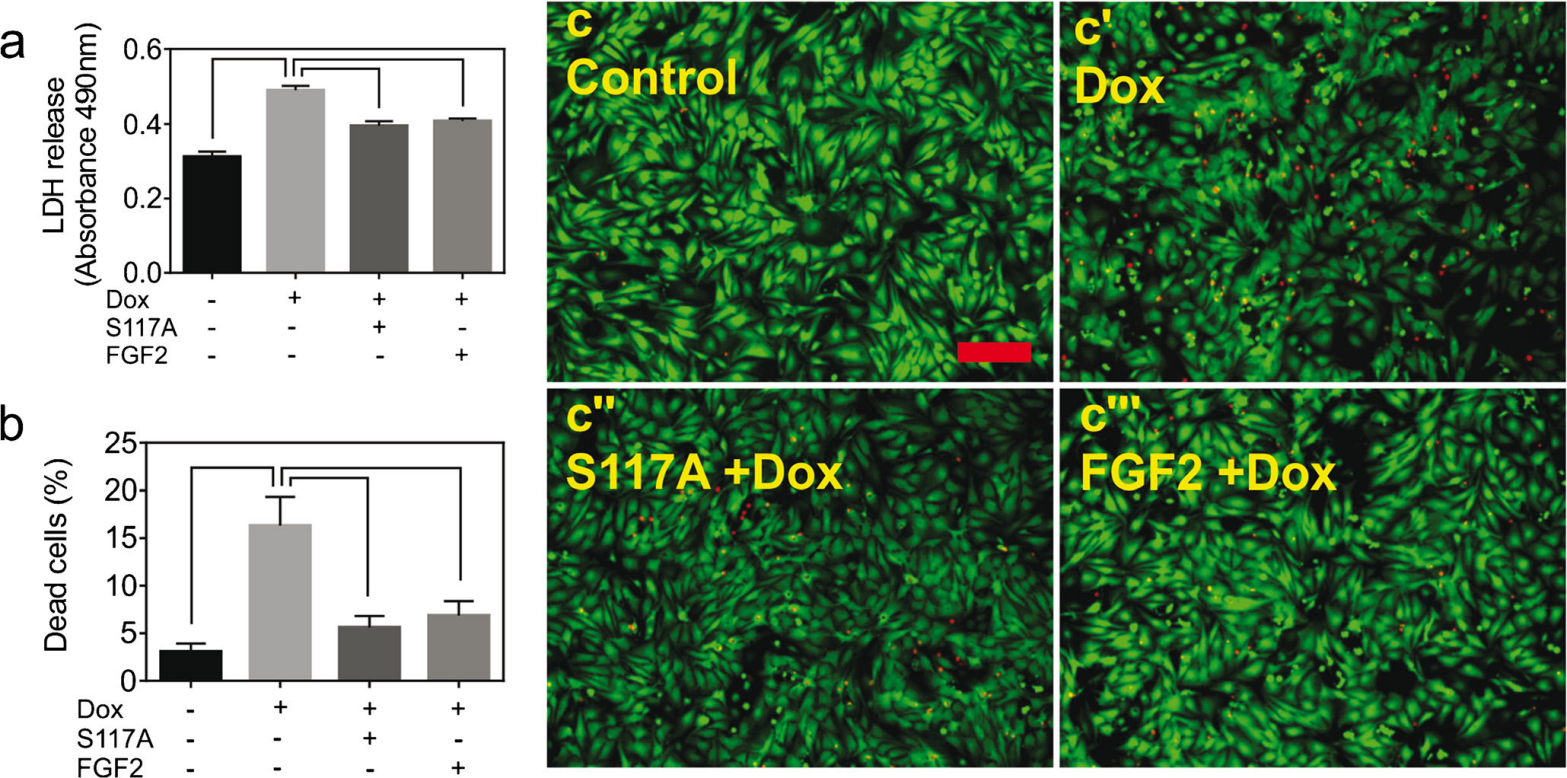
Non-mitogenic (S117A) FGF2 protects cardiomyocytes from Dox toxicity. (a) The effect of doxorubicin (Dox) on LDH released to the medium by cardiomyocytes, as well as the effect of pretreatment with S117A-FGF2, versus FGF2, on Dox-induced LDH release, as indicated. (b) The effect of Dox on the percentage of dead cells, as measured by the calcein-AM/ethidium homodimer assay; the effect of pretreatment with S117A-FGF2, or FGF2, on Dox-induced cell death is also shown. Representative fluorescence images are included in (c-c′″), where green or red stain live or dead cells, respectively. Brackets mark groups that differ significantly from each other (*n* = 4), in both (a) and (b)

We recently reported that primary cultures of neonatal rat cardiomyocytes, referred to as cardiomyocytes from now on, represent a good in vitro model to study multiple aspects of acute Dox-induced toxicity (Koleini et al. 2017). This model was used to examine the effect of pretreatment with S117A-FGF2 and FGF2 (used as positive control), on Dox-induced LDH release and cell death. As shown in Fig. 1(a), S117A-FGF2 was equally able as FGF2 in reducing cardiomyocyte plasma membrane damage compared to Dox, estimated by decreased LDH release. Using a Calcein-AM/ethidium homodimer assay, where live cells stain green (calcein-AM) and dead cells are stained red (ethidium homodimer), S117A-FGF2 was found to decrease the relative abundance of dead cells significantly Fig. 1(b, c–c″′).

We next examined the effect of S117A-FGF2 on a breast cancer cell line, MCF-7 cells. MCF-7 exposure to Dox for 24 h increased LDH release, an effect that was not prevented by S117A-FGF pre-incubation (Supplement, Fig. S1). Using an MTT assay as an estimate of cell number, we found that S117A-FGF2 had no effect on proliferation, unlike FGF2 that elicited a small but statistically significant increase in cell number (Fig. S1).

### The role of FGFR1 and ERK in S117A protection against dox-induced cardiomyocyte damage

**Fig. 2:**
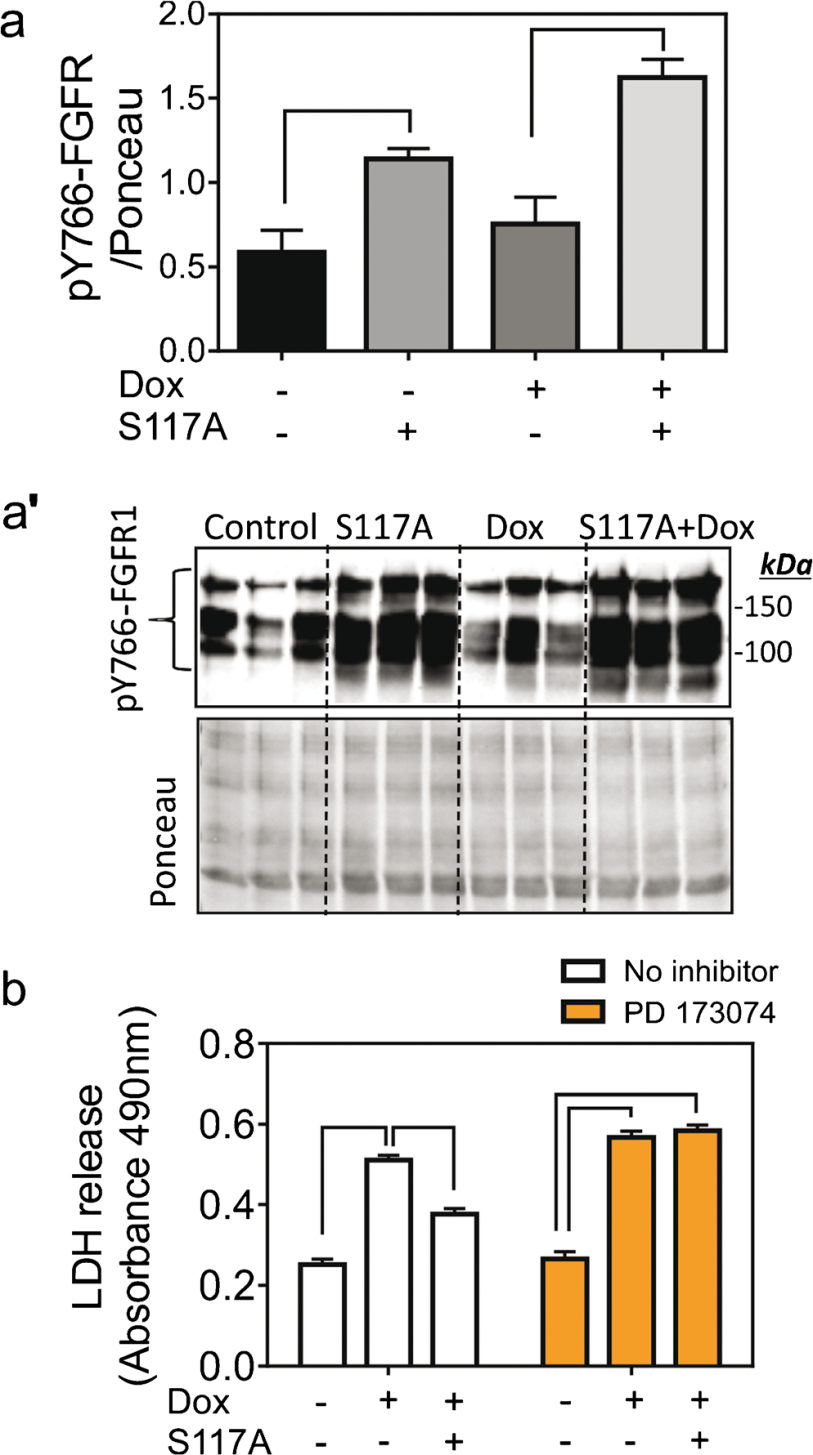
S117A-FGF2 requires FGFR1 to protect cardiomyocytes against Dox damage. (a) The effect of S117A-FGF2 on relative pY766-FGFR1 after 30-min cardiomyocyte stimulation and after Dox exposure for another 30 min. Brackets show groups significantly different from each other (*n* = 3). Images of the corresponding western blot probed for anti-pY766-FGFR1, or stained with Ponceau S are also included (a’). (b) The effect of PD173074 (FGFR1 inhibitor) on S117A-FGF2 protection from Dox-induced LDH release. Brackets point to groups that are significantly different from each other. In the presence of PD173074, S117A-FGF2 is unable to reduce Dox-induced LDH release

Fibroblast growth factor receptor-1 (FGFR1) is the main cardiomyocyte FGF2 receptor (Kardami et al. 1995; Liu et al. 1995). Upon ligand binding, FGFR1 dimerizes and becomes trans-phosphorylated on tyrosine (Y) 766 (Ornitz and Itoh 2015). As shown in Fig. 2(a, a′), S117A-FGF2 stimulation for 30 min significantly upregulated relative levels of pY766-FGFR1 compared to unstimulated cells. Increased pY766-FGFR1 levels in S117A-FGF2-treated cells persisted even after Dox exposure, Fig. 2(a, a’). Pre-incubation of cardiomyocytes with the specific FGFR1 inhibitor PD173074 abolished the S117A-FGF2-induced protection from Dox-induced LDH release (Fig. 2b).

**Fig. 3:**
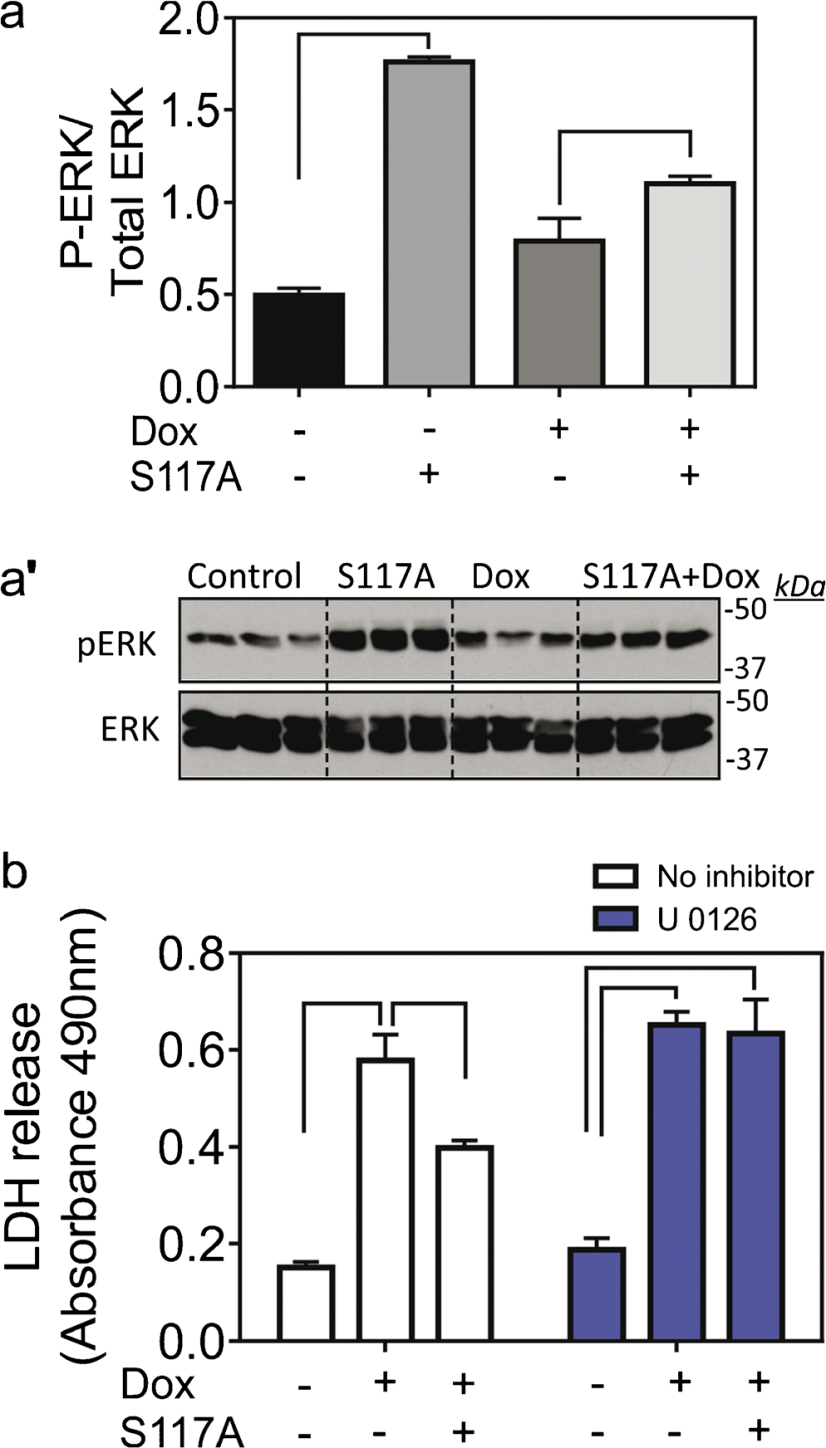
The S117A-FGF2 protection from Dox requires ERK activity. (a) The effect of S117A-FGF2 on pERK/ERK ratio after 30 min cardiomyocyte stimulation and after Dox exposure for another 30 min. Images of the corresponding western blots probed for anti-pERK or anti-ERK are also included (a′, *n* = 3). (b) The effect of UO126 (inhibitor of ERK activation) on S117A-FGF2 protection from Dox-induced LDH release. Brackets point to groups that are significantly different from each other. In the presence of UO126, S117A-FGF2 is unable to reduce Dox-induced LDH release

Several kinases associated with regulation of cardioprotection are known to be activated downstream of the FGF2/FGFR1 axis. These include ERK (or p44/42 MAPK), p38 and AKT (Katoh 2016). Exposure of cardiomyocytes to S117A-FGF2 for 30 min upregulated P-ERK, compared to unstimulated cells (Fig. 3a, a’). Phospho-ERK remained elevated in S117A-FGF2-treated cells even after incubation with Dox for 30 min (Fig. 3a, a’). The specific ERK inhibitor U0261 abolished S117A-FGF mediated cardiomyocyte protection against Dox (Fig. 3b). In addition, as shown in Fig. S2, both mitogenic FGF2 and S117-FGF2 upregulated cardiomyocyte P-p38 as well as P-AKT, after 60 min.

### The role of HO-1 and CK2 in S117A-FGF2 protection against Dox-induced cardiomyocyte damage

**Fig. 4:**
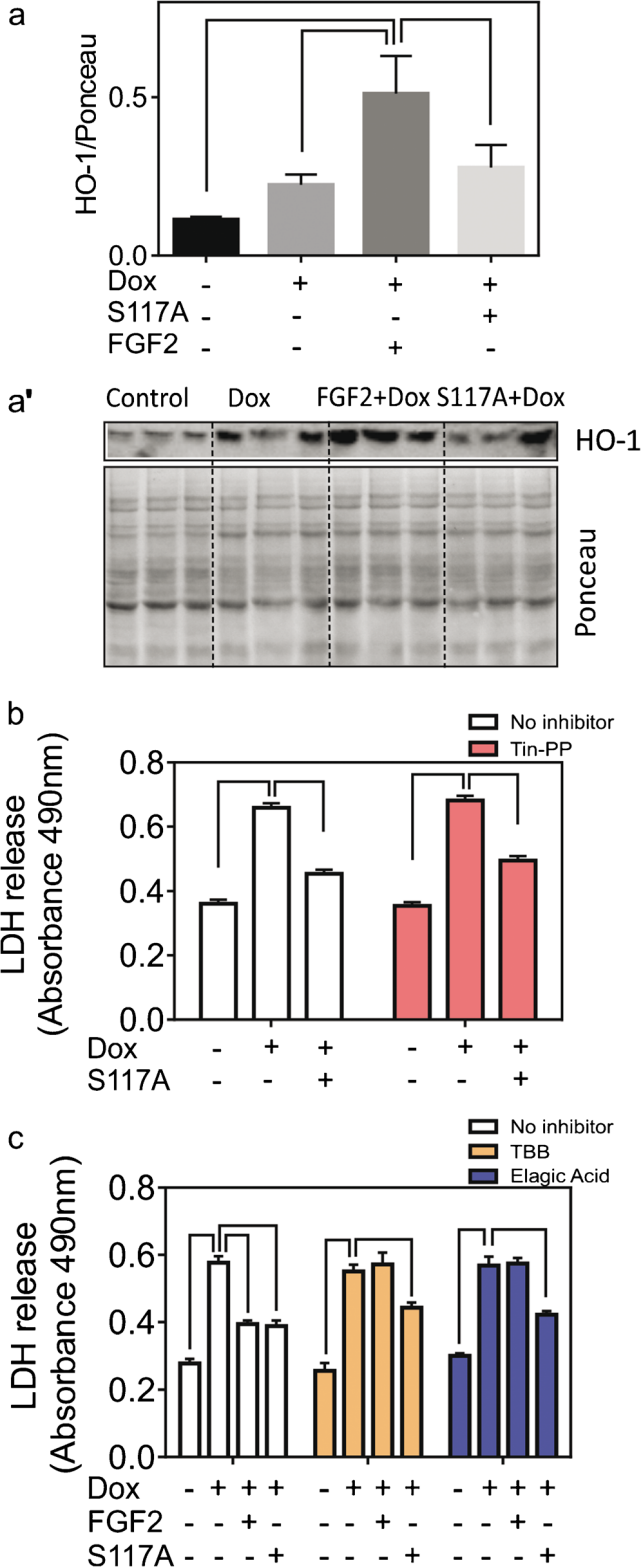
The S117A-FGF-2 protection from Dox does not require HO-1 or CK2 activity. (a) The effect of S117A-FGF2, versus FGF2, on relative HO-1 levels in cardiomyocytes treated with Dox, as estimated by western blotting. Images from the corresponding blot probed for HO-1, or stained with Ponceau S, are also included (a′). (b) The effect of Tin-PP (HO-1 inhibitor) on S117A-FGF2 protection from Dox-induced LDH release, as indicated. Inhibition of HO-1 does not prevent S117A-FGF2 protection. (c) The effect of two CK2 inhibitors, TBB or Elagic acid, on the protective effect of S117A-FGF2, or FGF2, against Dox-induced LDH release. In the absence of inhibitors, both S117A-FGF2 and FGF2, significantly reduce Dox-induced LDH release. In the presence of either inhibitor, FGF2 is no longer protective, while S117A-FGF2 retains significant protective ability. In all panels, brackets denote groups significantly different from each other

We have recently shown that FGF2 upregulated HO-1 in Dox-treated cardiomyocytes and that FGF2-induced cardiomyocyte protection from Dox was blocked by the HO-1 inhibitor Tin-protoporphyrin (Tin-PP) (Koleini et al. 2017). We have now examined the ability of S117A-FGF2 to upregulate HO-1 and the ability of Tin-PP to prevent S117A-FGF2 protection from Dox. As shown in Fig. 4(a, a’), while FGF2 elicited significant upregulation of the HO-1 protein, compared to control or Dox-treated myocytes, S117A-FGF2 had no effect. Furthermore, Tin-PP did not block S117A-FGF protection against Dox-induced LDH release (Fig. 4b), indicating that HO-1 was not required for S117A-FGF2 protection.

We also examined the role of CK2, a likely upstream activator of HO-1 upregulation (Kim et al. 2012), in mediating protection by S117A-FGF2, in comparison to FGF2, from Dox-induced cardiomyocyte damage. Two CK2 inhibitors were used, TBB and Ellagic acid. These inhibitors blocked cardiomyocyte protection by FGF2 but not S117A-FGF2 (Fig. 4c).

### Effects of S117A on accumulation of reactive oxygen species and oxidized phospholipids

**Fig. 5:**
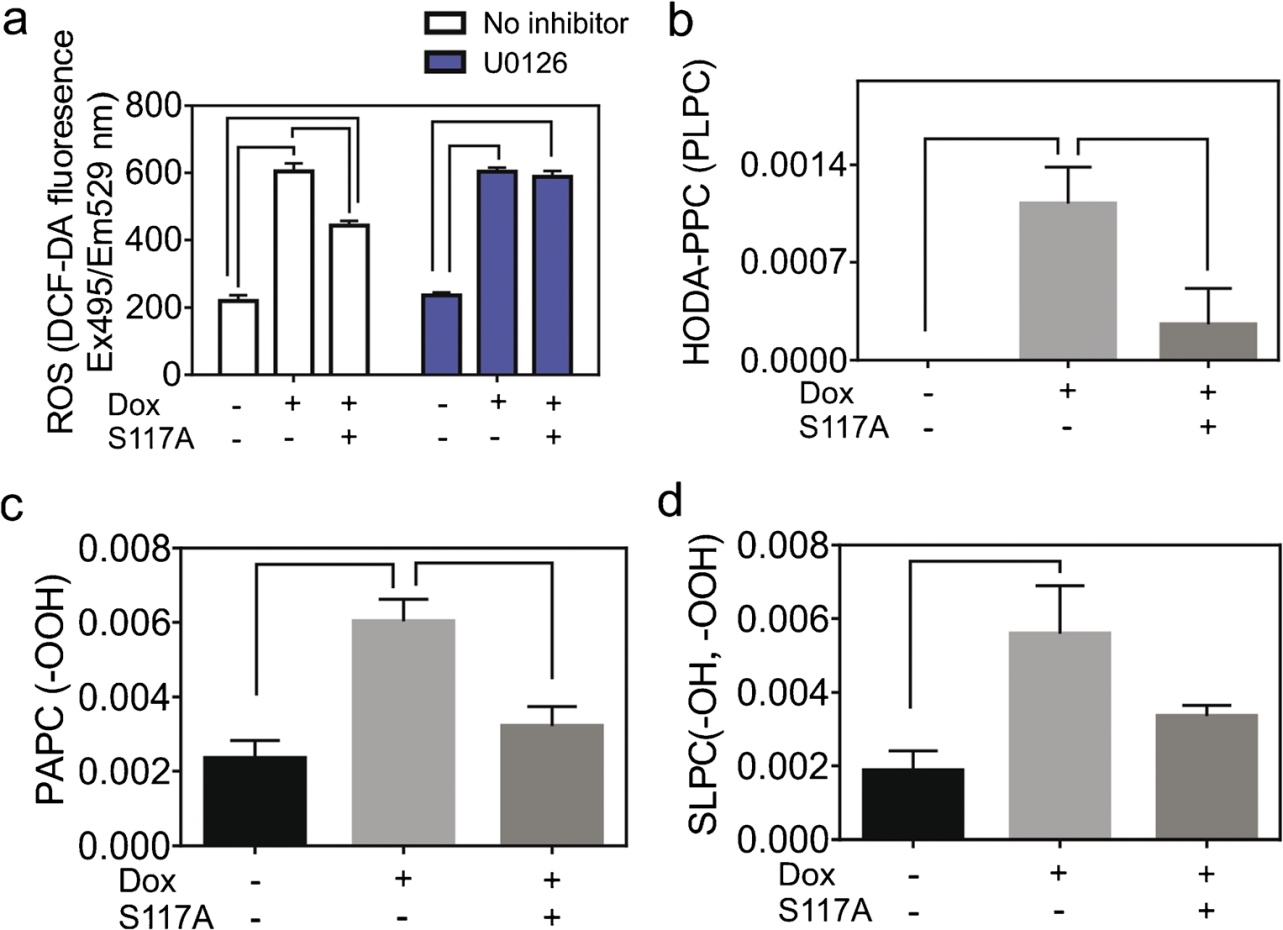
S117A-FGF2 prevents Dox-induced ROS accumulation and oxidation of phosphatidylcholines. Neonatal rat cardiomyocytes were treated with Dox in the presence and absence of S117A-FGF-2 pre-treatment. **a** The effect of S117A-FGF2 on Dox-induced Dox upregulation as measured by the DCF-DA assay. **b**–**d** Levels of oxidized phosphatidylcholine species (HODA-PPC (PLPC); PAPC (–OOH); SLPC (–OH, –OOH)) measured by LC/MS/MS and normalized for protein concentration. The *Y*-axis in panels **b**–**d** shows the amount (ng) of OxPC per μg cardiomyocyte protein. In all panels, brackets denote groups significantly different from each other (*n* = 3 independent analyses)

Excessive reactive oxygen species (ROS) production is a major contributor to Dox-induced cardiomyocyte toxicity (Damiani et al. 2016). As shown in Fig. 5(a), S117A-FGF2 attenuated the Dox-induced accumulation of ROS in cardiomyocytes, measured by the fluorescence intensity of 2′,7’dichlorodihydrofluorescein diacetate (DCF-DA). Inhibition of ERK blocked the S117A-FGF2- mediated decreases in ROS production but did not appear to decrease Dox-induced ROS (Fig. 5a).

Increased ROS causes oxidation of DNA, proteins and peroxidation of lipids. It has been shown previously that oxidized phospholipid (OxPC) molecules are increased in cardiac tissue in response to Dox treatment and correlate with left ventricular dysfunction (Zeglinski et al. 2014). We hypothesized that a cardioprotective agent should decrease relative levels of Dox-induced oxidized phospholipids. We measured the most abundant oxidation products of polyunsaturated phospholipid species present in cardiomyocytes: oxidized forms of three phosphatidylcholines (a class of phospholipids): 1-palmitoyl-2-arachidonoyl-*sn*-glycero-3-phosphocholine (PAPC) 1-palmitoyl-2-linoleoyl-sn-glycero-3-phosphocholine (PLPC), and 1-stearoyl-2-linoleoyl-*sn*-glycero-3-phosphocholine (SLPC).

Shown in Fig. 5(b), Dox-induced formation of a fragmented PLPC (HODA-PPC, 9-hydroxy-12-oxo-10-dodecenoic acid), which was not detectable in controls, an effect that was significantly attenuated by S117A-FGF2. Dox also increased relative levels of PAPC (-OOH) and SLPC (-OH, -OOH); S117A-FGF2 significantly attenuated the Dox-induced increase in the former, Fig. 5(b), but not the latter oxidized phosphatidylcholine (Fig. 5 c, d).

**Fig. 6:**
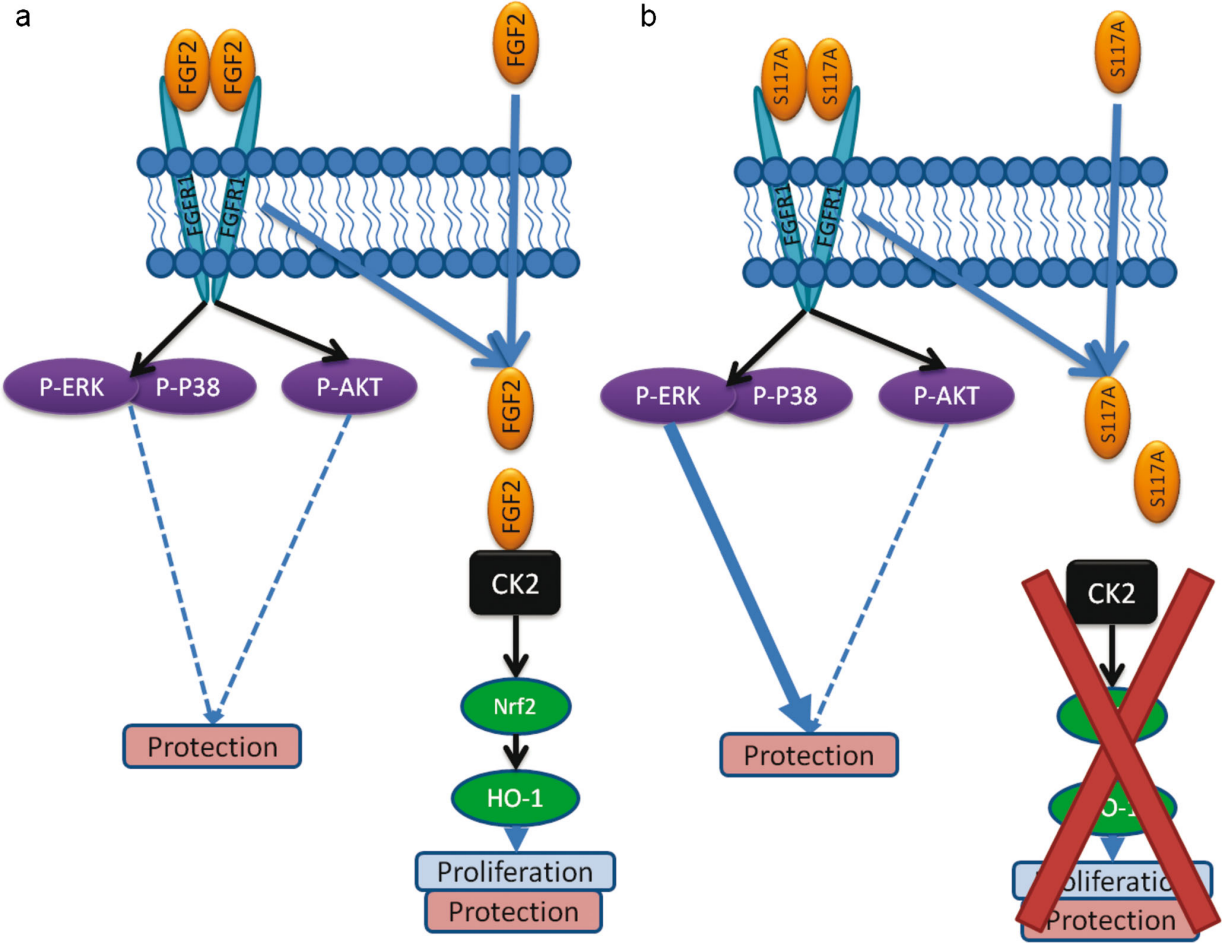
Diagramatic representation of the proposed signaling mechanisms mediating FGF2 versus S117A-FGF2 protection from Dox-induced damage. **a** Signaling by mitogenic FGF2, which activates FGFR1 and downstream kinases implicated in the modulation of cytoprotection, including ERK, p38 and AKT; the dotted lines indicate potential effects based on general literature but not studied here. Internalized FGF2 (via FGFR1-dependent or FGFR1-independent mechanism), interacts with and activates CK2 and CK2-dependent pathways (Nrf2, HO-1) that promote proliferation and proliferation-linked cytoprotection. **b** Signaling by S117A-FGF2, which lacks the ability to activate CK2 and the CK2-dependent effects on proliferation. S117-FGF2 retains the ability to activate FGFR1 and downstream kinases (ERK, p38 and  AKT); of these, ERK was shown here to be required for S117-FGF2 protection in Dox-treated myocytes

In additional analyses, as shown in the Supplement (Fig. S3), Dox was found to significantly upregulate PAPC (-OH) and SLPC (-epoxy, -keto) but these changes remained unaffected by S117A-FGF2A. Dox, with or without S117A-FGF2, had no statistically significant effect on PLPC (-keto), PLPC (-OOH), KODA-PPC (PLPC) and SONPC (SLPC). A schematic representaion of the signaling pathways potentially involved in S117A-FGF2 versus FGF2 in cardiomyocyte protection against Dox is shown in Fig. 6.

## Discussion

The main findings of this study are (1) a mutated non-mitogenic form of FGF2, S117A-FGF2, protects cardiomyocytes against acute Dox-induced toxicity, (2) S117A-FGF2 protection is contingent on FGFR1/ERK activation, (3) CK2 and HO-1 are not required for S117A-FGF2 protection and (4) S117A-FGF2 protects from Dox-induced upregulation of certain oxidized phosphatidylcholines.

### Non-mitogenic FGF2 protects cardiomyocytes from Dox-induced toxicity

Our previous studies, documenting the protective potential of FGF2 isoforms against Dox-induced cardiomyocyte injury and cell death, validated the use of LDH release as an overall indicator of cellular damage; the pattern from the LDH release data was paralleled by measurement of % dead cells (live-dead assay) and by the overall reduction in ROS, as well as several other markers associated with cell death (Koleini et al. 2017). The present study used LDH release as a representative end-point to assess and document protection by non-mitogenic S117A-FGF2 from Dox-induced damage. Results from the LDH release assay were paralleled by the relative reduction in: percentage of dead cells; overall ROS production; reduction in specific OxPC species. Taken together, our results demonstrated, for the first time, that S117A-FGF2 protected cardiomyocyte from Dox-induced cellular damage and death, to a similar extent as FGF2, at least in the acute setting (24 h) of our experiment. In a previous study, S117A-FGF2 was found to attenuate the Dox-induced decline in developed pressure of isolated perfused mouse hearts, although no evidence of Dox-induced cellular injury/death was detectable during the 2 h of perfusion (Sontag et al. 2013).

Regarding the corresponding mechanism of protection, summarized in Fig. 6, and as shown previously in different cardiac injury models (Jiang et al. 2002), protection by S117A-FGF2, as by FGF2, required the activation of FGFR1; indeed, as shown here, S117A-FGF2 promoted significant Y766-FGFR1 phosphorylation and a specific FGFR1 inhibitor blocked S117A-FGF2 protection. ERK is activated downstream of FGFR1 and is considered a cardiac pro-survival kinase (Hausenloy et al. 2005). Our results show that non-mitogenic FGF2 depends on FGFR1/ERK signaling for protection against acute Dox toxicity.

A class of chemotherapeutic drugs (e.g., Sunitinib) relies on the inhibition of tyrosine kinase receptors such as FGFR1 (Chu et al. 2007); S117A-FGF2 that depends on FGFR1 activity for cardioprotection, would therefore not be expected to be cardioprotective in patients receiving these categories of drugs but would still be useful in anthracycline-based treatments. Localized administration, or gene-therapy based approaches, such as, for example, systemic administration of AAV9-expressing S117A-FGF2, could deliver S117A-FGF2 to the heart and induce protection.

CK2 has been associated with the proliferation and survival of normal, as well as rapidly proliferating cancer cells (Trembley et al. 2009). FGF2 activates nuclear CK2 that then targets nucleolin; this sequence of events is important to the mitogenic effect of FGF2 (Bailly et al. 2000). FGF2-triggered activation of CK2 requires internalization of FGF2. In addition, it has been shown that FGF2 can be internalized via FGFR1-dependent and FGFR1-independent (Syndecan-4) pathways (Tkachenko et al. 2004). However, it is not as yet determined if FGFR1 activation is needed for FGF2 internalization/CK2 activation in cardiomyocytes. It would appear that CK2 activity is also required for the pro-survival effect of mitogenic FGF2 in our model, since two CK2 inhibitors, TBB and Ellagic acid, eliminated FGF2 protection, as shown here. It is therefore intriguing that S117A-FGF2, which cannot activate CK2, retains pro-survival effects against Dox. This was further validated by our findings that CK2 inhibitors did not block S117A-FGF2 protection. Thus, a CK2-independent pathway, activated in parallel to, or downstream of FGFR1/ERK, mediated protection by the non-mitogenic S117A-FGF2. Detailed description of the FGF2 versus S117-FGF2-triggered pathways awaits further studies using systems biology approaches. It would seem, however, that neither AKT nor p38 kinases are responsible for the differences between mitogenic and non-mitogenic FGF2, since they were similarly activated by both.

CK2 can activate the Nrf2 transcription factor and upregulate its downstream target HO-1 in chondrocytes (Kim, Song, Chung and Park 2012). It is likely that CK2 is also required for the FGF2-induced HO-1 upregulation in Dox-treated cardiomyocytes (Koleini et al. 2017). Catabolic end-products elicited by the action of HO-1 exert “antioxidant, anti-apoptotic, and immune-modulating effects, leading to overall cytoprotective and beneficial functions” (Salerno et al. 2017) for non-malignant tissues. On the other hand, in the case of cancer, HO-1 is pro-tumorigenic and causes resistance to anti-cancer drug treatments; inhibition or modulation of HO-1-generating pathways is beneficial in the treatment of leukemia (Salerno et al. 2017). As we showed here, S117A-FGF2 did not upregulate HO-1 and its ability for acute cardiomyocyte protection against Dox was not blocked by HO-1 inhibition. This reinforces the notion that S117A-FGF2 is an attractive candidate to provide acute cardiac protection from Dox-treatment, as it would not affect cancer cell proliferation and survival via the CK2/HO-1 axis. Our study that showed that S117A-FGF2 did not promote survival or proliferation of the breast cancer cell line MCF-7 supports the therapeutic potential of S117A-FGF2. In addition, our previous studies demonstrated that S117-FGF2, unlike FGF2, is incapable of stimulating cardiomyocyte DNA synthesis, measured by the incorporation of bromodeoxyuridine in the genomic DNA (Jiang, Srisakuldee, Soulet, Bouche and Kardami 2004).

### S117A prevents Dox-induced upregulation of ROS and OxPC

One of the main mechanisms contributing to Dox cardiotoxicity is increased ROS accumulation that causes DNA and mitochondrial damage, iron toxicity and lipid peroxidation (Koleini and Kardami 2017). We found that ERK inhibition prevented S117A-FGF2-mediated downregulation of Dox-induced ROS accumulation and thus conclude that activation of ERK by S117A-FGF2 (via FGFR1) is a critical step toward its protective antioxidant effects.

Hydroxy radical OH^•^ formed during Dox-induced oxidative and nitrosative stress can cause severe oxidative damage to proteins and DNA and can promote lipid peroxidation. Lipid peroxidation is a chain reaction, mediated by free radicals (mainly OH^•^), leading to structural damages to unsaturated fatty acids of the phospholipids (Damiani et al. 2016). There is increasing understanding of the importance of oxidized phospholipids in the development of cardiovascular disease (Allen et al. 2013; Salomon 2012). It has been shown that oxidized phospholipids can alter signaling pathways in the cells, induce formation of mitochondrial permeability transition pores leading to cytochrome C release and apoptosis and act as a signal on the plasma membrane to promote binding of leukocytes (Salomon 2012). Oxidation can cause a vast number of changes in the structure of fatty acids forming various types of structurally and functionally different biomolecules. In chemotherapy-induced cardiomyopathy OxPC have been shown to correlate with myocardial toxicity, left ventricular dysfunction and cell death (Bordun et al. 2015; Goyal et al. 2016; Koleini et al. 2014). Given the biological activity of OxPC, they are not only bystanders of cellular ROS production but also have toxic effects towards cardiomyocytes in a different scenario, reperfusion injury (Ganguly et al. 2018; White et al. 2013; White et al. 2015).

To our knowledge, the present study shows for the first time that Dox increased several OxPC-containing phospholipids in cardiomyocytes, including PAPC(-OOH), SLPC(-OH, -OOH) and HODAPPC. Protection by S117A-FGF2 was associated with attenuation or prevention of Dox-induced upregulation of these oxidized phosphatidylcholines. It is reasonable to expect that the S117A-FGF2-induced decreases in overall ROS production contributed to decreased production of the OxPC measured and that decreased ox-phospholipid levels contributed to protection. It is intriguing that S117-FGF2 appeared to be selective in protecting against increases of only some of the OxPC species that were upregulated by Dox. The reason for this selectivity is unclear at present but could be explained by hypothesizing that several different signaling pathways and/or different subcellular sites are involved in the oxidation of the various PC species and only some of them are accessible to S117-FGF2 signal transduction.

We evaluated the protective effects of non-mitogenic FGF2 in an in vitro model of Dox cardiotoxicity. Further experiments are required to validate the protective effects of S117A-FGF2 against Dox in vivo. In addition, short- versus long-term effects of S117A-FGF2 treatment in vivo need to be investigated.

In conclusion, we showed non-mitogenic FGF2 maintains the ability for acute cardioprotection from Dox cardiomyocyte toxicity while lacking characteristics that are undesirable in a cancer environment, such as the potential for CK2 activation and upregulation of HO-1. Our findings reinforce the notion that non-mitogenic FGF2 should be considered as an acute cardioprotective or prophylactic therapy against Dox cardiotoxicity.

## Electronic supplementary material

Figure S1 S117A-FGF2 does not protect MCF-7 cells from Dox-induced cell death or damage (LDH release), and does not stimulate MCF-7 cell proliferation. Panel a-a” shows representative images of MCF-7 cells subjected to Dox, in the abcence or presence of S117A-FGF2 pre-treatment, stained based on the live/dead, Calcein-AM/Ethidium homodimer assay. A similar abundance of red (dead) cells can be seen rehardless of S117A-FGF2 in Dox-treated cells. Panel b shows the absence of an effect of S117A-FGF2 on Dox-induced LDH release. Panel c shows the effect of S117A-FGF2, and FGF2, on MCF-7 cell proliferation, as estimated by the MTT assay. In al lpanels, bracket mark groups that are significantly different from each other. (PNG 637 kb)

High Resolution (TIF 25.6 mb)

Figure S2 Neonatal rat cardiomyocytes were stimulated with S117A-FGF2 or FGF2 (10 ng/ml) for 60 min. Both S117A-FGF2 and FGF2 were able to increase phosphorylation of AKT (tyrosine 473) and P38 (panels a-a″′). Phospho-ERK was also upregulated at this time point, included for comparisons. The experiment was done with *n* = 4 and the brackets in the corresponding graph show significant differences between the groups. (PNG 329 kb)

High Resolution (TIF 19.4 mb)

Figure S3 The graphs show the effect of Dox, in the absence or presence of S117A-FGF2, on the oxidation of additional phosphatidylcholine species, not included in Fig. 5. Brackets point to groups that are significantly different from each other (*n* = 3). The Y axis shows the amount (ng) of OxPC per μg cardiomyocyte protein. (PNG 1102 kb)

High Resolution (TIF 13.7 mb)
