# Supplementary figures and images for: Non-mitogenic FGF2 protects cardiomyocytes from acute doxorubicin-induced toxicity independently of the protein kinase CK2/heme oxygenase-1 pathway
Source: Cell Tissue Res. 2018 Aug 29;374(3):607–17. doi: 10.1007/s00441-018-2905-z (PMC6267702; doi:10.1007/s00441-018-2905-z)

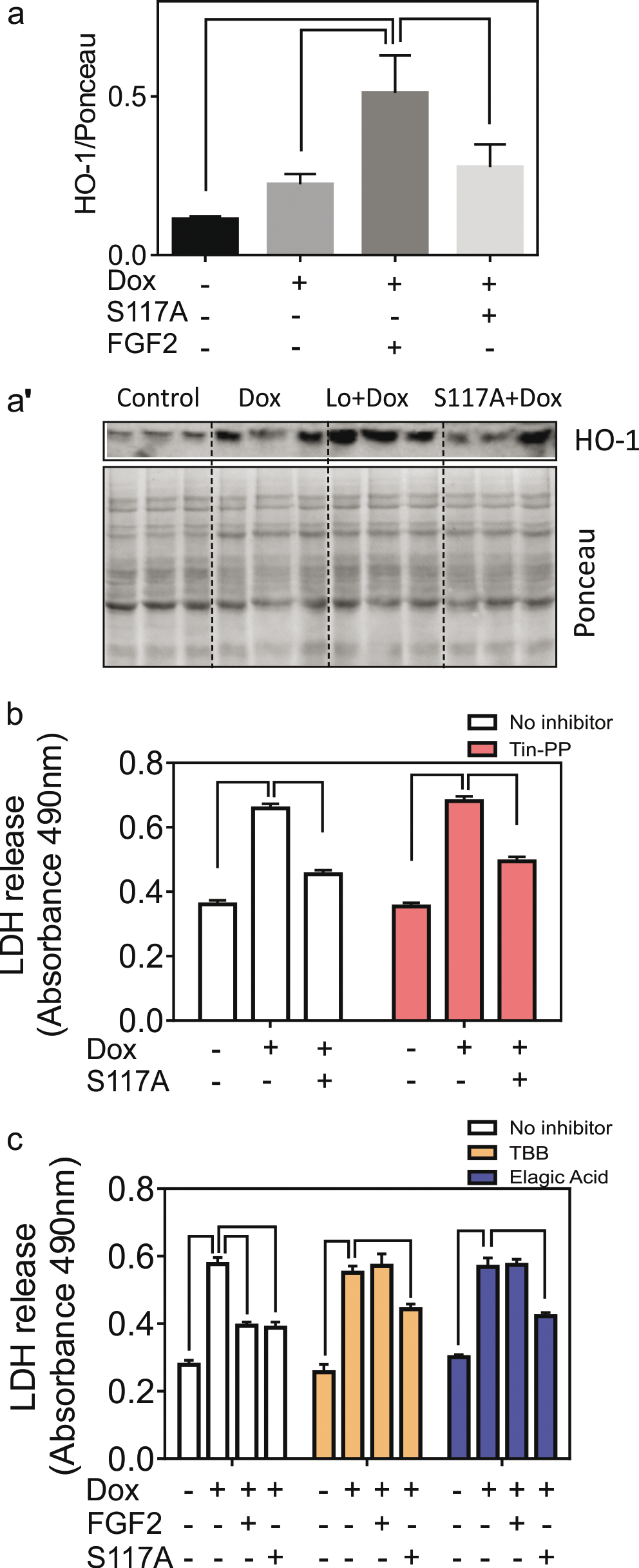

Supplement: Supplementary file 1 — S117A-FGF2 does not protect MCF-7 cells from Dox-induced cell death or damage (LDH release), and does not stimulate MCF-7 cell proliferation. Panel a-a” shows representative images of MCF-7 cells subjected to Dox, in the abcence or presence of S117A-FGF2 pre-treatment, stained based on the live/dead, Calcein-AM/Ethidium homodimer assay. A similar abundance of red (dead) cells can be seen rehardless of S117A-FGF2 in Dox-treated cells. Panel b shows the absence of an effect of S117A-FGF2 on Dox-induced LDH release. Panel c shows the effect of S117A-FGF2, and FGF2, on MCF-7 cell proliferation, as estimated by the MTT assay. In al lpanels, bracket mark groups that are significantly different from each other. (PNG 637 kb) [file 441_2018_2905_Fig7_ESM.png]

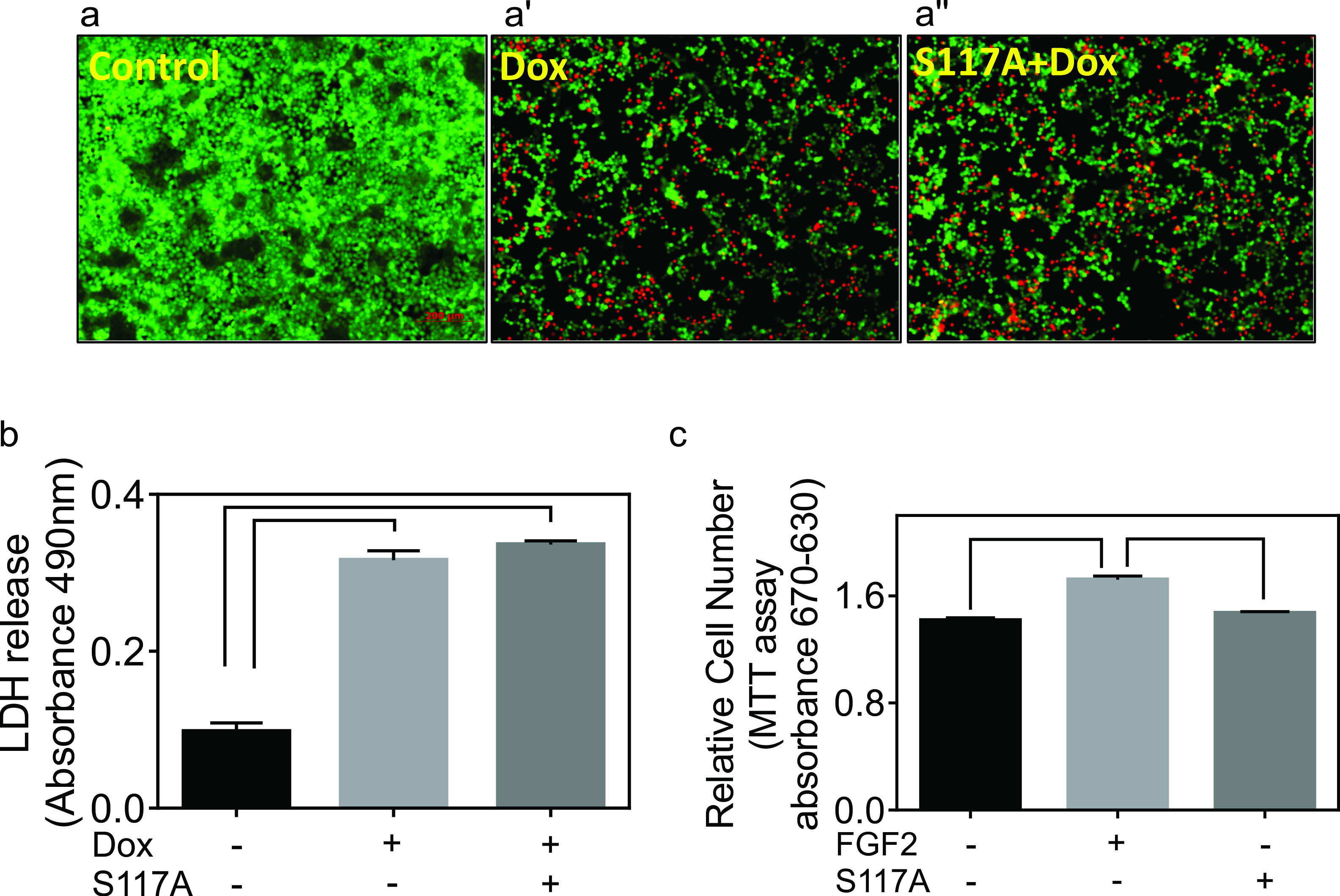

Supplement: Supplementary file 2 — High Resolution (TIF 25.6 mb) [file 441_2018_2905_MOESM1_ESM.tif]

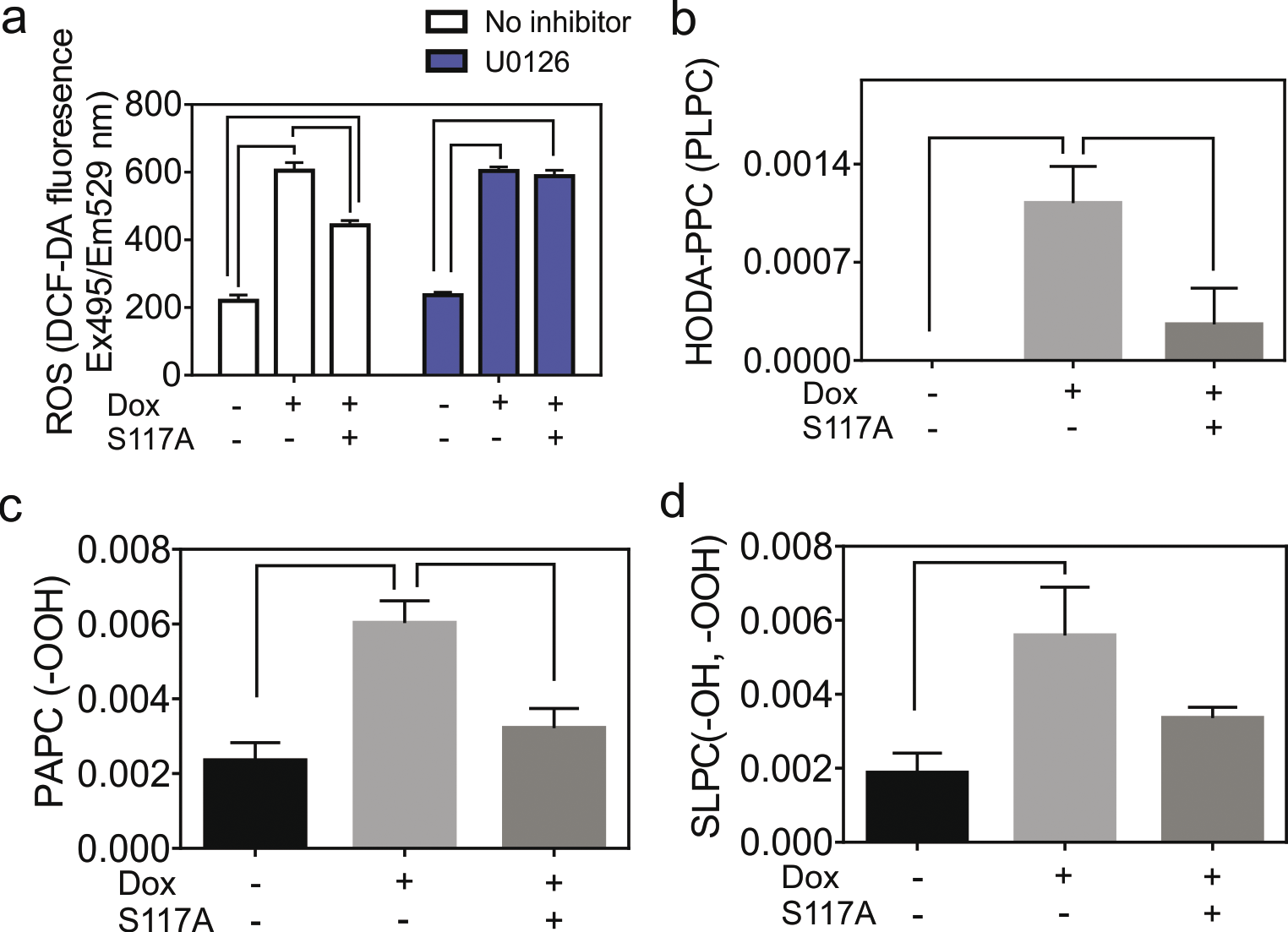

Supplement: Supplementary file 3 — Neonatal rat cardiomyocytes were stimulated with S117A-FGF2 or FGF2 (10 ng/ml) for 60 min. Both S117A-FGF2 and FGF2 were able to increase phosphorylation of AKT (tyrosine 473) and P38 (panels a-a″′). Phospho-ERK was also upregulated at this time point, included for comparisons. The experiment was done with n = 4 and the brackets in the corresponding graph show significant differences between the groups. (PNG 329 kb) [file 441_2018_2905_Fig8_ESM.png]

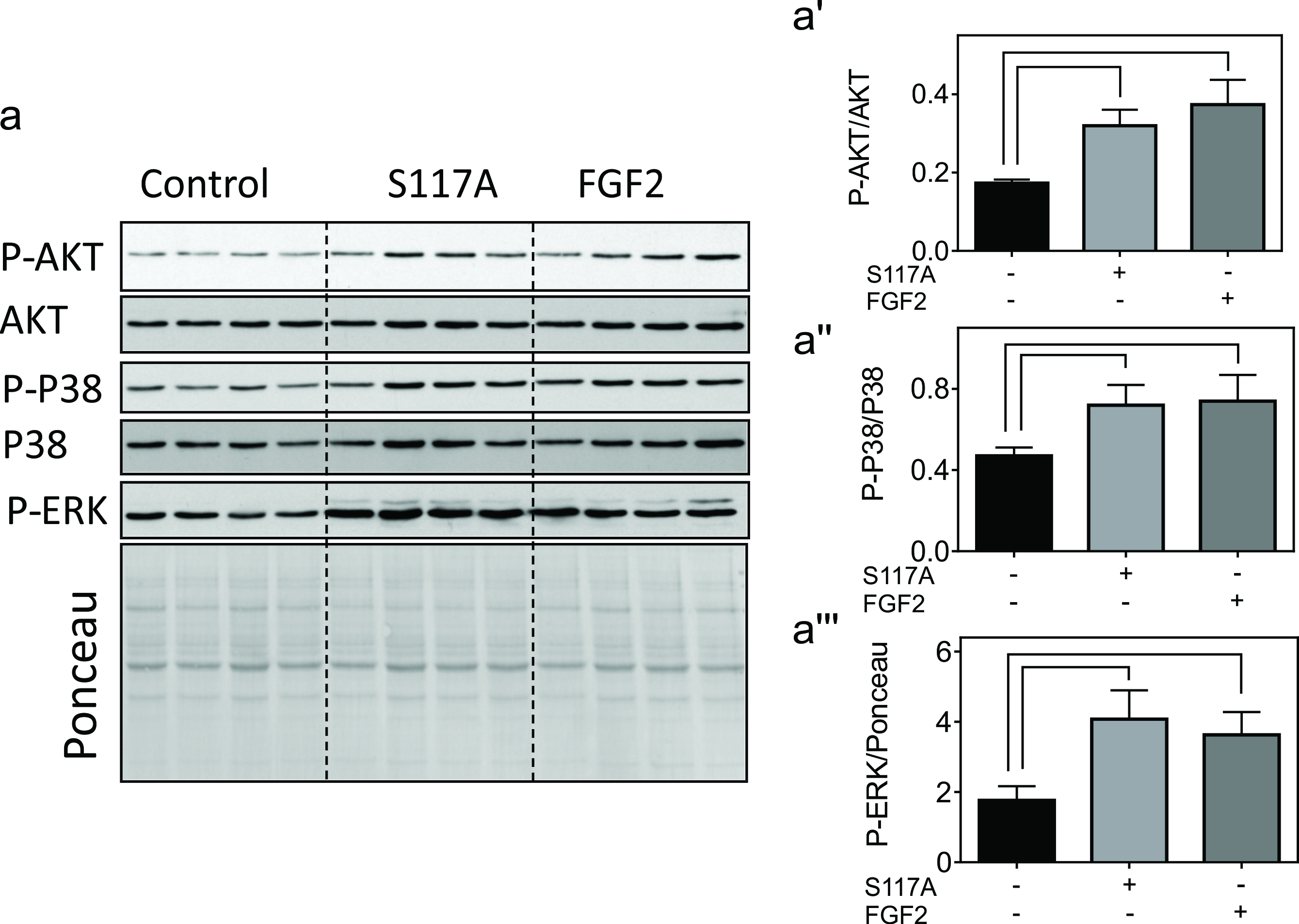

Supplement: Supplementary file 4 — High Resolution (TIF 19.4 mb) [file 441_2018_2905_MOESM2_ESM.tif]

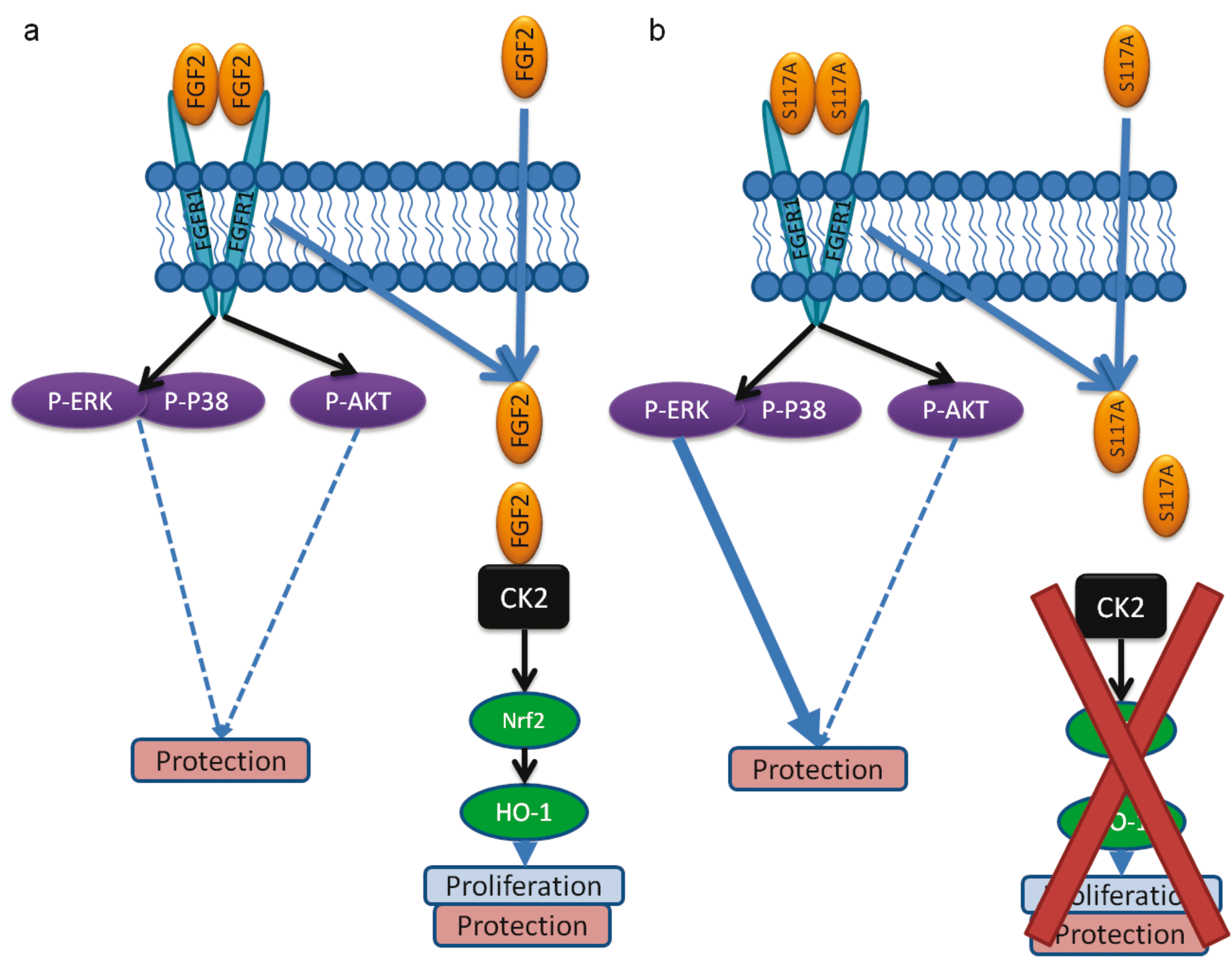

Supplement: Supplementary file 5 — The graphs show the effect of Dox, in the absence or presence of S117A-FGF2, on the oxidation of additional phosphatidylcholine species, not included in Fig. 5. Brackets point to groups that are significantly different from each other (n = 3). The Y axis shows the amount (ng) of OxPC per μg cardiomyocyte protein. (PNG 1102 kb) [file 441_2018_2905_Fig9_ESM.png]

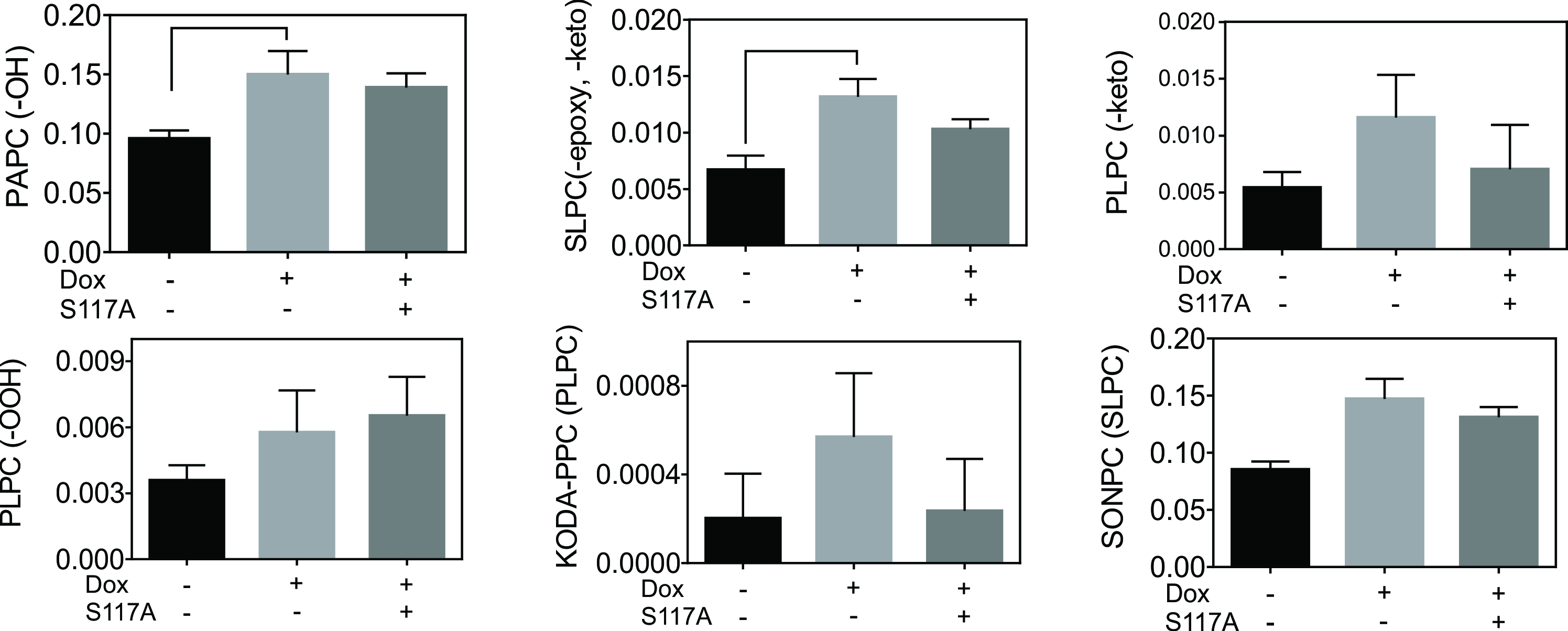

Supplement: Supplementary file 6 — High Resolution (TIF 13.7 mb) [file 441_2018_2905_MOESM3_ESM.tif]
